# Wind of Change: Brexit and European Rehabilitation

**DOI:** 10.15171/ijhpm.2017.133

**Published:** 2017-11-08

**Authors:** Jorge Hugo Villafañe, Pablo Herrero, Pedro Berjano

**Affiliations:** ^1^IRCCS Fondazione Don Carlo Gnocchi, Milan, Italy.; ^2^Universidad San Jorge, Zaragoza, Spain.; ^3^IRCCS Istituto Ortopedico Galeazzi, Milan, Italy.

## Dear Editor,

The historic British referendum on June 23, 2016 has perhaps placed in the hands of our European Union (EU) a couple of stunning cards to play in order to improve our position in the international arena. The Brexit decision was very controversial as the percentage of people that voted for Brexit was not much higher than those that voted to remain in the EU (51.9% voted to leave whilst 48.1% voted to remain). In addition, there were great differences between different territories (eg, 62% of Scotland voted to remain) and age ranges(eg, 73% of 18-24 year-olds and 62% among 25-34s voted to remain).^1^ A recent debate on the impact of Brexit on research was held on May 8, 2017 at the Royal Institution in London, and organised in collaboration with EuroScience and the European Academy. The debate clearly demonstrated on one hand the lack of preparedness of the science community, as a whole, to face the challenges ahead and, on the other, the fear of the loss of influence of UK researchers on the European scene was palpable during the discussions.^2^ Several comments immediately following the British vote for Brexit focused on the negative effects both in general terms and in individual sectors, such as that of scientific research.^3,4^ Immigration, is the issue on which those seeking Brexit have focused most. ‘Freedom of Movement’ is a core principle of the EU, enshrined in its treaties, alongside the other three basic freedoms of movement of goods, capital and services and is a concrete manifestation of EU citizenship.^5^ Many benefits to UK public health from EU legislation would likely be retained after a Brexit. The health service faces severe financial constraints and appears to lurch from crisis to crisis, with leaving the EU likely to exacerbate many problems including staffing issues across the whole sector. However, new scientific developments and digital technology offer societies everywhere massive and unprecedented opportunities for improving health. It is vital for the country that the National Health Service (NHS) is able to adopt these discoveries and see them translated into improved patient care and population health, but also that the United Kingdom benefits from its capabilities and strengths in these areas.^6^ It would have to pay to participate in EU structures. Post-Brexit UK might be able to participate in research, as do Switzerland, Norway, and Israel, among others, by buying into the programme but it would have no input to policy. Moreover, its participation would depend on what the EU would allow.^7^ The EU budget for 2014–2020 for research, development, and innovation is estimated at €120 billion, including €74.8 billion in the current Framework Programmes, known as Horizon 2020.^8^ UK health research received €119.9 million in 2015 from Horizon 2020 programs, equal to 22.2%, 20.7% and 20.2% of the EU-13, EU-15 and EU-28, respectively^3^; whether or not they can continue to participate in such funding programs after Brexit will need to be renegotiated.

How scientific development will be distributed in the next years depends on the knowledge, skill, motivation and organization of research teams, but other factors are equally important. How research funding is distributed among disciplines, areas, countries or teams is of key importance in the evolution of the scientific landscape. In the last years, EU funding for research has been uneven. Even so, some countries were still able to contribute to research while receiving insufficient funding, despite being less advanced in innovation and research.^4^

At least two factors that are not related to scientific value can influence the distribution of research funding, thus contributing to deepen the gap among more and less scientifically developed countries and disciplines of science (countries with *non-scientific advantage* would attract more funding, which in turn would increase their scientific value compared to countries or disciplines attracting less funding, which finally would increase the *scientific advantage* to further attract funding). The first of these factors is language. English speaking countries (or countries with a large penetration of English as a second language, such as the Nordic and Scandinavian area) have shown to receive more funding than predominantly non-English speaking countries. Though their superior performance could be partially a consequence of overall scientific superiority, the difference in researchers’ ability to write in good English cannot be overlooked as a source of financing inequality.

From a linguistic point of view, although English is the first language in the scientific world, this is not so for many EU citizens. If we want science to have an impact that is evenly dispersed among the European population, the unfair language bias must be removed. The official language for countries applying for funding applications should be considered, especially in projects with social impact in countries where the population does not speak English, as this involves a double effort. First, applying in English and later in translating all the material or content for the language of every country, with all its social and cultural implications. If the United Kingdom is no longer able to access EU funding, their portion of the pie becomes available for other countries in Europe to use in their own research. Countries such as Italy and Spain with strong research initiatives and infrastructure and great research potential could take advantage of the newly available funding more so than countries such as Germany, the Netherlands, and France that already receive ample amounts of funding. In this scenario, removing a strong competitor with natural language advantage could potentially allow other countries to emerge. The second non-scientific factor influencing the ability to attract research funding is priority. Financing agencies establish European funding priorities based on consensus. The process to consensus includes the opinion of administrators, politicians, and citizens; also scientists and research institutions are requested to provide feedback on their perception of priority, but the exact mechanisms how consensus is obtained is not clearly defined. Our prediction is that the distribution of scientific research funding developed under the EU-27 will be altered and that possibly we will see major impacts from Italy and Spain (or other countries now accounting for small allocations of funds). It is also likely that countries with large allocations of research funds (Germany, France, the Netherlands) will increase their participation.

The idea that any country can act entirely independently in a globalized world, or should do, is a dangerous fantasy. Understanding challenges and opportunities that rehabilitation researchers may face once the United Kingdom leaves the EU will help to prepare rehabilitation researchers in the EU for the next decade.^3,4^

## Ethical issues

Not applicable.

## Competing interests

Authors declare that they have no competing interests.

## Authors’ contributions

Concept development: JHV; Writing: JHV, PH, PB; Literature search: JHV, PH; Critical review: JHV, PH, PB.

## Authors’ affiliations

^1^IRCCS Fondazione Don Carlo Gnocchi, Milan, Italy. ^2^Universidad San Jorge, Zaragoza, Spain. ^3^IRCCS Istituto Ortopedico Galeazzi, Milan, Italy.
